# Sudden Death in Childhood

**Published:** 1905-07-29

**Authors:** 


### SUDDEN DEATH IN CHILDHOOD.

Sudden death in adults is of course common enough, though few adults die suddenly without having experienced some premonitions of the insecurity of their lives. But in childhood death not infrequently appears without any announcement whatever. Such a visitation naturally strikes home with an overwhelming force, and, by reason of its inexplicability, may easily throw upon innocent people imputations far from warranted. It is important, therefore, to recognise that the tenure of life, in infancy especially, may be interrupted by a variety of circumstances besides the common lethal processes demonstrable in the dead house, or by the microscope. A few observations recorded by Dr. Charles Macalister[1] sufficiently point this moral, and we may employ them as a text for a brief review of the present state of knowledge in the matter.

Speaking generally, the children most liable to sudden death are those whose margin of vitality is reduced, although the reduction may be totally insufficient to justify suspicions of a fatal issue. This reduction may depend upon some unrecognised and unrecognisable congenital malformation, or upon atelectasis pulmonum, or upon some chronic, though perhaps slight, nutritional disorder. As instancing the occurrence of sudden death in the first of these conditions we may record the case of a girl of four years whose fæces at birth were discharged by the vagina, the anal canal being absent. An artificial anus was successfully made, but there remained a minute recto-vaginal fistula for the relief of which a second operation was performed. The patient died suddenly during convalescence from an attack of measles, and the only discoverable lesion after death was a congenital absence of one kidney, and cystic disease of the other.

Atelectasis may be congenital or acquired. In either case it is found in puny and ill-nourished infants, especially those born prematurely, and may bring life sharply to an end after a brief period of cyanosis and dyspnœa. Marasmic children often die quite suddenly, sometimes from a pulmonary collapse which their weakness does not enable them to overcome, sometimes it would seem from nothing more definite than an exhaustion of their vital resources.

Sudden death in weakly children is sufficiently discomposing to all concerned, but the matter assumes a far more painful aspect when the victim appears to be in the most robust health. Foremost in this category come the deaths of infants whose only morbid feature after death consists in an enlargement of the thymus gland. This "Thymic asthma" as it is called is not at all uncommon. The subjects of it are generally breast-fed children whose excellent appearance would seem to bespeak a faultless condition of health. The end may come quite suddenly, or may be attendant upon some apparently insignificant malady, and is supposed (without good reason, as it seems to us) to be due to pressure of the enlarged gland upon the pneumogastric nerves. Very occasionally plump but rickety infants die in the spasm of laryngismus stridulus, and though it is the rule for the spasms to increase gradually in their severity, in exceptional cases the first attack is a severe or even a fatal one.

It is no rarity for infants to die unexpectedly in convulsions. These children are generally ill-nourished and often rickety, although sufficient organic lesions to account for the fatal result are seldom forthcoming at the autopsy. Such cases supply the bulk of those brought dead to a children's hospital, a succession of convulsions terminating in death while the patient is being hurried to the institution.

Finally we have the group of cases whose prime features are a sudden onset of illness associated with a gradually rising fever, and death in a few hours. It is with cases of this sort that Dr. Macalister is most concerned in the paper above referred to. This paper contains a remarkable series of cases which form a good illustration of the type. A boy, previously in good health, complained one day of headache; towards evening he was seized with violent vomiting and diarrhœa, gradually passed into a condition of extreme lividity, and died in three hours. Two years later in the same institution another boy complained of headache at 7 p.m. one evening. In the morning of the next day he was found to be very ill, and died at 7 a.m., or twelve hours after the onset of symptoms. The patient, like the other, looked as though he had been strangled, his whole body being cyanosed, and the veins in the neck extraordinarily turgid. At the autopsy the only lesion found was a small area of pneumonic consolidation, the lungs being generally congested and exuding a brownish fluid. Four months later a third lad was taken suddenly with vomiting and fever one night, and was found dead in bed the following morning, his appearance being of the same livid character as that presented by the two previous cases. No autopsy was permitted in this case.

These three cases are good examples of a type which almost certainly depends upon an extremely virulent infection of the blood by micro-organisms or of the system by the absorption of toxins of some kind. A similar sequence of events may occasionally be observed where the surface of the body has been severely and extensively burnt, and at a period sufficiently remote to exclude the operation of shock; also after septic operations upon bones, such as those undertaken for the relief of chronic middle-ear disease. It is much to be regretted that no bacteriological examination of the blood appears to have been made in Dr. Macalister's cases, for it is highly probable that such an examination would have disclosed the cause of the disasters. Similar in rationale, no doubt, are the malignant types of scarlet fever, measles, small-pox, and other exanthemata.

Asphyxia, whether from overlying, or from aspiration of food into the larynx, accounts for a certain number of sudden and unexpected deaths, and in this class should be included the rare instances of death from the paroxysms of whooping cough. This contingency is very remote, except in the case of very young and feeble victims of the disease, in whom the exhaustion following the paroxysm may cause a period of apnœa prolonged till death, unless artificial means of respiration are resorted to in time.

Such are the chief varieties of sudden death in childhood, leaving out of account those which occur in the course of serious organic disease such as morbus cordis and pleural effusions.

[1] Pediatrics, May 1905.

